# Universal adversarial examples and perturbations for quantum classifiers

**DOI:** 10.1093/nsr/nwab130

**Published:** 2021-07-22

**Authors:** Weiyuan Gong, Dong-Ling Deng

**Affiliations:** Center for Quantum Information, Institute for Interdisciplinary Information Sciences (IIIS), Tsinghua University, Beijing 100084, China; Center for Quantum Information, Institute for Interdisciplinary Information Sciences (IIIS), Tsinghua University, Beijing 100084, China; Shanghai Qi Zhi Institute, Shanghai 200232, China

**Keywords:** quantum machine learning, quantum classifiers, adversarial examples, measure concentration, quantum no-free-lunch theorem

## Abstract

Quantum machine learning explores the interplay between machine learning and quantum physics, which may lead to unprecedented perspectives for both fields. In fact, recent works have shown strong evidence that quantum computers could outperform classical computers in solving certain notable machine learning tasks. Yet, quantum learning systems may also suffer from the vulnerability problem: adding a tiny carefully crafted perturbation to the legitimate input data would cause the systems to make incorrect predictions at a notably high confidence level. In this paper, we study the universality of adversarial examples and perturbations for quantum classifiers. Through concrete examples involving classifications of real-life images and quantum phases of matter, we show that there exist universal adversarial examples that can fool a set of different quantum classifiers. We prove that, for a set of *k* classifiers with each receiving input data of *n* qubits, an *O*(ln [*k*]/2^*n*^) increase of the perturbation strength is enough to ensure a moderate universal adversarial risk. In addition, for a given quantum classifier, we show that there exist universal adversarial perturbations, which can be added to different legitimate samples to make them adversarial examples for the classifier. Our results reveal the universality perspective of adversarial attacks for quantum machine learning systems, which would be crucial for practical applications of both near-term and future quantum technologies in solving machine learning problems.

## Teaser

The existence of universal adversarial examples and perturbations for quantum classifiers has been demonstrated both analytically and numerically, revealing a crucial vulnerability aspect for quantum machine learning with near-term and future technologies.

## INTRODUCTION

Machine learning, or more broadly artificial intelligence, has achieved dramatic success over the past decade [1,2] and a number of problems that were notoriously challenging, such as playing the game of Go [3,4] or predicting protein structures [5], have been cracked recently. In parallel, the field of quantum computing [6] has also made remarkable progress in recent years, with the experimental demonstration of quantum supremacy marked as the latest milestone [7,8]. The marriage of these two fast-growing fields gives birth to a new research frontier—quantum machine learning [9–11]. On the one hand, machine learning tools and techniques can be exploited to solve difficult problems in quantum science, such as quantum many-body problems [12], state tomography [13], topological quantum compiling [14], structural and electronic transitions in disordered materials [15], non-locality detection [16] and classification of different phases of matter and phase transitions [17–24]. On the other hand, new quantum algorithms running on quantum devices also possess the unparalleled potentials to enhance, speed up or innovate machine learning [9–11]. Notable examples along this direction include the Harrow-Hassidim-Lloyd algorithm [25], quantum principal component analysis [26], quantum generative models [27–29] and quantum support vector machines [30], etc. Without a doubt, the interaction between machine learning and quantum physics will benefit both fields [11].

In classical machine learning, it has been shown that classifiers based on deep neural networks are rather vulnerable in adversarial scenarios [31,32]: adding a tiny amount of carefully crafted noises, which are even imperceptible to human eyes and ineffective to traditional methods, into the original legitimate data may cause the classifiers to make incorrect predictions at a notably high confidence level. A celebrated example that clearly showcases the vulnerability of deep learning was observed by Szegedy *et al.* [33], where an image of a panda will be misclassified as a gibbon after adding an imperceptible amount of noise. The crafted input samples that would deceive the classifiers are called adversarial examples. Now, it is widely believed that the existence of adversarial examples is ubiquitous in classical machine learning—almost all learning models suffer from adversarial attacks, regardless of the input data types and the details of the neural networks [31,32]. More recently, the vulnerability of quantum classifiers has also been studied, sparking a new research frontier of quantum adversarial machine learning [34–39]. In particular, Lu *et al.* [34] explored different adversarial scenarios in the context of quantum machine learning and have demonstrated that, with a wide range of concrete examples, quantum classifiers are likewise highly vulnerable to crafted adversarial examples. This emergent research direction is growing rapidly, attracting more and more attentions across communities. Yet, it is still in its infancy and many important issues remain unexplored.

**Figure 1.**
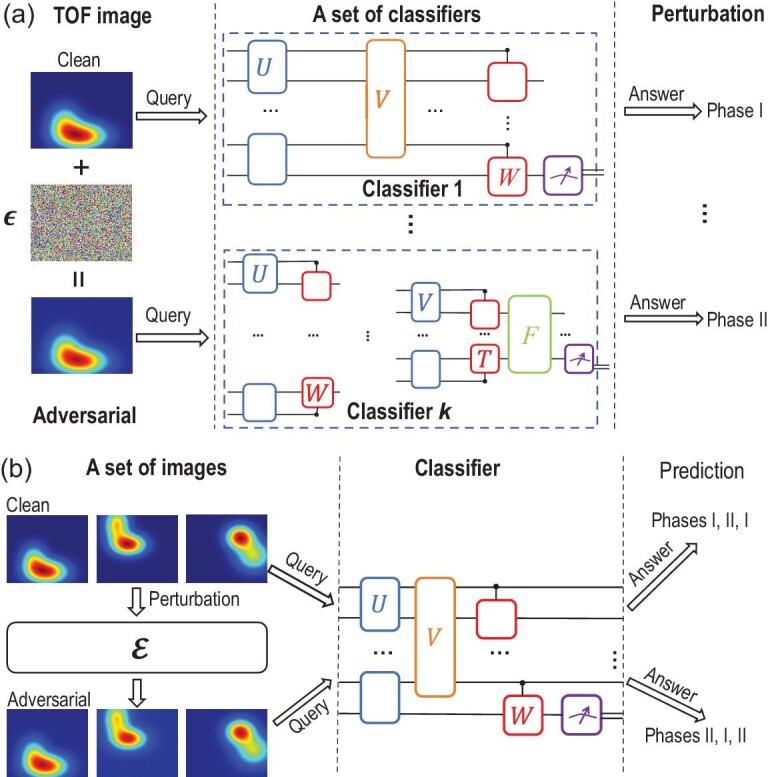
A schematic illustration of universal adversarial examples and perturbations. (a) Universal adversarial examples: a set of quantum classifiers can be trained to assign phase labels to different time-of-flight images, which can be obtained directly in cold atom experiments. Adding a small amount of carefully crafted noise to a certain image could make it become a universal adversarial example (i.e., the new crafted image could deceive all the classifiers in the set). (b) Universal adversarial perturbations: adding the same carefully constructed noise to a set of images could make them all become adversarial examples for a given quantum classifier.

In this paper, we consider such an issue concerning the universality of adversarial examples and perturbations for quantum classifiers (see Fig. 1 for the schematic illustration). We ask two questions: (i) whether there exist universal adversarial examples that could fool a set of different quantum classifiers and (ii) whether there exist universal adversarial perturbations, which when added to different legitimate input samples could make them become adversarial examples for a given quantum classifier. Based on extensive numerical simulations and analytical analysis, we give affirmative answers to both questions. For (i), we prove that, by exploring the concentration of measure phenomenon [40], an *O*(ln [*k*]/2^*n*^) increase of the perturbation strength is enough to ensure a moderate universal adversarial risk for a set of *k* quantum classifiers with each receiving input data of *n* qubits. For (ii), we prove that, based on the quantum no-free-lunch theorem [41,42], the universal adversarial risk is bounded and approaches unity exponentially fast as the number of qubits for the quantum classifier increases. We carry out extensive numerical simulations on concrete examples involving classifications of real-life images and quantum phases of matter to demonstrate how to obtain universal adversarial examples and perturbations in practice.

## UNIVERSAL ADVERSARIAL EXAMPLES

To begin with, we first introduce some concepts and notations. Consider a classification task in the setting of supervised learning, where we assign a label *s* ∈ *S* to an input data sample $\rho \in \mathcal {H}$, with *S* being a countable label set and $\mathcal {H}$ the set of all possible samples. The training set is denoted as $\mathcal {S}_N=\lbrace (\rho _1,s_1),\ldots ,(\rho _N,s_N)\rbrace$, where $\rho _i\in \mathcal {H}$, *s*_*i*_ ∈ *S* and *N* is the size of the training set. Essentially, the task of classification is to learn a function (called a hypothesis function) $h:\mathcal {H}\rightarrow S$ that, for a given input $\rho \in \mathcal {H}$, outputs a label *s*. We denote the *ground truth* function as $t:\mathcal {H}\rightarrow S$, which gives the true classification for any $\rho \in \mathcal {H}$. For the purpose in this paper, we suppose that after the training process the hypothesis function matches the ground truth function on the training set, namely *h*(ρ) = *t*(ρ) for all $\rho \in \mathcal {S}_{N}$. We consider a set of *k* quantum classifiers $\mathcal {C}_1,\ldots ,\mathcal {C}_k$ with corresponding hypothesis functions *h*_*i*_ (*i* = 1, …, *k*) and introduce the following definitions to formalize our results.

Definition 1. **Definition 1.** We suppose that the input sample ρ is chosen from $\mathcal {H}$ according to a probability measure μ and $\mu (\mathcal {H})=1$. For *h*_*i*_, we define $\mathcal {E}_i=\lbrace \rho \in \mathcal {H}|h_i(\rho )\ne t(\rho )\rbrace$ as the misclassified set, and the *risk* for $\mathcal {C}_i$ is denoted as $\mu (\mathcal {E}_i)$.

Definition 2. **Definition 2.** Consider a metric over $\mathcal {H}$ with the distance measure denoted as *D*( · ). Then the ε expansion of a subset $\mathcal {H}^{\prime }\subseteq \mathcal {H}$ is defined as $\mathcal {H}^{\prime }_\epsilon =\lbrace \rho |D_{\rm {min}}(\rho ,\mathcal {H}^{\prime })\le \epsilon \rbrace$, where $D_{\rm {min}}(\rho ,\mathcal {H}^{\prime })$ denotes the minimum distance between ρ and any $\rho ^{\prime }\in \mathcal {H}^{\prime }$. In the context of adversarial learning, a perturbation within distance ε added to the legitimate input sample $\rho \in \mathcal {E}_{i,\epsilon }=\{\rho' | D_{\rm {min}}(\rho',\mathcal{E}_i)\leq \epsilon \}$ can shift it to some misclassified one for the quantum classifier $\mathcal {C}_i$. Hence, we define the adversarial risk for $\mathcal {C}_i$ as $\mu (\mathcal {E}_{i,\epsilon })$. Similarly, the universal adversarial risk for a set of *k* quantum classifiers is defined as $R=\mu (\mathcal {E}_\epsilon )$, where $\mathcal {E}_\epsilon =\bigcap _{i=1}^k\mathcal {E}_{i,\epsilon }$ denotes the set of universal adversarial samples.

For simplicity and convenience, we focus on $\mathcal {H}={\rm SU}(d)$ (the special unitary group) with the Hilbert-Schmidt distance *D*_HS_(ρ, ρ^′^) and Haar probability measure [43]. We mention that the input data ρ can be either classical or quantum in general. We treat both cases on the same footing since we can always encode the classical data into quantum states. We also note that any input state could be prepared by acting a unitary transformation on a certain initial state (e.g. the |00⋅⋅⋅0〉 state) and hence the classification of quantum states is in some sense equivalent to the classification of unitary transformations. Now, we are ready to present one of our main results.

**Theorem 1.** Consider a set of *k* quantum classifiers $\mathcal {C}_i,\, i=1,\ldots ,k$, and let $\mu (\mathcal {E})_{{\rm min}}$ be the minimum risk among $\mu (\mathcal {E}_i)$. Suppose that ρ ∈ SU(*d*) and that a perturbation ρ → ρ^′^ occurs with *D*_HS_(ρ, ρ^′^) ≤ ε. Then we can ensure that the universal adversarial risk is bounded below by *R*_0_ if

(1) \begin{equation*} \epsilon ^2\ge \frac{4}{d}\ln\! {\bigg [\frac{2k}{\mu (\mathcal {E})_{{\rm min}}(1-R_0)}\bigg ]}. \end{equation*}

*Proof.* We give the main idea and intuition here. The full proof is a bit technically involved and thus left to the online supplementary material. The first step is to prove that, for a single quantum classifier $\mathcal {C}_i$, we can ensure that its adversarial risk is bounded below by *R*_0, *i*_ if $\epsilon ^2\ge \frac{4}{d}\ln {[\frac{2}{\mu (\mathcal {E}_i)(1-R_{0,i})} ]}.$This can be done by exploring the concentration of the measure phenomenon for SU(*d*) equipped with the Haar measure and Hilbert-Schmidt metric [35]. Next, we use De Morgan’s laws in set theory to deduce that $\mu (\mathcal {E}_\epsilon )\ge 1-k+ \sum _{i=1}^k\mu (\mathcal {E}_i)$. In the last step, we choose *R*_0, *i*_ = (*k* − 1 + *R*_0_)/*k* and replace $\mu (\mathcal {E}_i)$ by $\mu (\mathcal {E})_{\text{min}}$ to increase ε a little bit for each $\mathcal {C}_i$. This leads to Equation (1) and completes the proof.

The above theorem implies that, for a set of *k* quantum classifiers with each receiving input data of *n* qubits (thus *d* = 2^*n*^), an *O*(ln [*k*]/2^*n*^) increase of the perturbation strength would guarantee a moderate universal adversarial risk lower bounded by *R*_0_. As *n* increases, the lower bound of ε approaches zero exponentially. In other words, an exponentially small adversarial perturbation could result in universal adversarial examples that can deceive all *k* classifiers with constant probability. This is a fundamental feature of quantum classifiers in high-dimensional Hilbert space due to the concentration of the measure phenomenon, independent of their specific structures and the input datasets.

Although the above theorem indicates the existence of universal adversarial examples in theory, it is still unclear how to obtain these universal examples in practice. To deal with this issue, in the following we provide concrete examples involving classifications of handwritten digit images and quantum phases with extensive numerical simulations. We mention that, in the classical adversarial machine learning literature, universal adversarial examples have also been shown to exist in real applications. For instance, in [44] it is shown that an attacker can fool (such as dodging or impersonation) a number of the state-of-the-art face-recognition systems by simply wearing a pair of carefully crafted eyeglasses. For our purpose, we consider a set of eight quantum classifiers with different structures, labeled by numbers from 1 to 8. The classifiers 1 and 2 are two quantum convolutional neural networks (QCNNs) [45] and classifiers 3–8 are other typical multi-layer variational quantum circuits with depths from five through ten. The detailed descriptions of these quantum classifiers are given in the online supplementary material.

**Figure 2.**
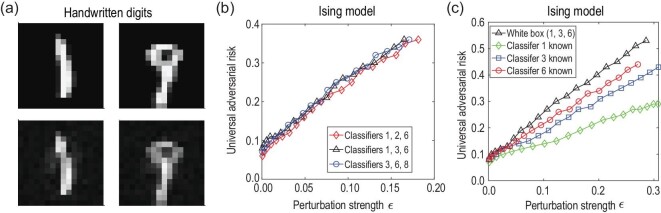
Numerical results on universal adversarial examples. In this figure, the adversarial examples are obtained through the qBIM algorithm with step size α = 0.02. (a) Illustrating samples: the clean (first row) and the corresponding universal adversarial handwritten digit images (second row) that can deceive all eight quantum classifiers. (b) The universal adversarial risk as a function of the perturbation strength ε for different subsets of the classifiers in classifying the ground states of the one-dimensional transverse field Ising model. Here, we consider the white-box attack scenario and the universal adversarial risk defined as the ratio of test samples that deceive all three classifiers in each subset. (c) Results for attacking a subset of classifiers consisting of the first, third and sixth classifiers in classifying the ground states of the Ising model, under a white-box black-box hybrid setting. Here, we assume that only one of the classifiers is known to the attacker. For comparison, the black curve with triangles plots the result for the white-box attack case. For more details, see the online supplementary material.

The first example we consider is the classification of handwritten digit images in the MNIST dataset [46], which is a prototypical testbed for benchmarking various machine learning scenarios. This dataset consists of gray-scale images of handwritten digits from 0 through 9, with each of them containing 28 × 28 pixels. We reduce the size of the images to 16 × 16, so that we can simulate the learning and attacking process of the quantum classifiers with moderate classical computational resources. We use amplitude encoding to map the input images into quantum states and the cross-entropy as the loss function for training and adversarial attacking. After training, we use the quantum-adapted basic iterative method (qBIM) [47] to obtain the adversarial examples. The details of the training and adversarial attacking process are provided in the online supplementary material. We mention that our quantum classifiers can achieve comparable training and validation accuracy as for classical classifiers. In Fig. 2(a), we display two universal adversarial examples for digits 1 and 9, which can deceive *all* eight quantum classifiers at a high confidence level. Notably, these universal adversarial examples only differ from the original legitimate ones slightly and they can be easily identified by human eyes. In fact, the fidelity between the adversarial and legitimate samples is about $96\%$, which is fairly high given that the Hilbert dimension involved is not very large (*d* = 256 for this case).

The above discussion concerns the vulnerability of quantum classifiers in classifying classical data (images). Yet, unlike classical classifiers that can only take classical data as input, quantum classifiers can also directly classify quantum data (states) produced by quantum devices. This is a notable distinction between quantum and classical classifiers. To demonstrate the existence of universal adversarial examples for quantum classifiers in classifying quantum data, we consider classifying the ground state of the one-dimensional (1D) transverse field Ising model:

(2) \begin{equation*} H_{\text{Ising}}=-\sum _{i=1}^{L-1}\sigma _i^z\sigma _{i+1}^z-J_x\sum _{i=1}^{L}\sigma _i^x. \end{equation*}

Here *J*_*x*_ denotes the strength of the transverse field and $\sigma _i^x$ and $\sigma _i^z$ are the Pauli matrices for the *i*th spin. This Hamiltonian maps to free fermions via a Jordan-Wigner transformation and is exactly solvable. Its ground state features a quantum phase transition at *J*_*x*_ = 1, between paramagnetic phase with *J*_*x*_ > 1 and ferromagnetic phase with 0 < *J*_*x*_ < 1. We consider classifying these two different phases by the eight quantum classifiers mentioned above, with the ground state as input data. We sample the Hamiltonian with varying *J*_*x*_ from 0 to 2 and compute their corresponding ground states. These quantum states with their corresponding labels form the dataset required.

In Fig. 2(b), we consider three subsets of quantum classifiers in classifying the ground states of *H*_Ising_, under the white-box attack setting (namely the attacker has full information about the learned model and the learning algorithm). We find that universal adversarial examples indeed exist for classifying quantum states, regardless of the internal structures of the classifiers. As the perturbation strength ε increases, the universal adversarial risk increases roughly linearly with ε. With a perturbation strength ε = 0.18, we find that $37\%$ of the test samples could become universal adversarial examples for each subset of the classifiers. In Fig. 2(c), we consider a white-box black-box hybrid scenario, where the attacker knows only the full information about one classifier in the subset and does not have any information about the rest. The motivation behind this consideration is to study the transferability of universal adversarial examples. From Fig. 2(c), we find that even with limited partial information, the adversary is still able to create universal adversarial examples, indicating a notable transferability property of these examples. The universal adversarial risk also increases linearly with ε, but it is noticeably smaller than that for the white-box case. This is consistent with the intuition that the more information the attacker has, the easier it is to create adversarial examples.

## UNIVERSAL ADVERSARIAL PERTURBATIONS

In the above discussion, we demonstrated, with both theoretical analysis and numerical simulations, that there exist universal adversarial examples that could deceive a set of distinct quantum classifiers. We now turn to the second question and show that there exist universal adversarial perturbations that can be added to different legitimate samples and make them adversarial to a given quantum classifier $\mathcal {C}$. Without loss of generality, we may consider a unitary perturbation $\hat{\epsilon }:\mathcal {H}\rightarrow \mathcal {H}$ as means of adversarial attack for all input samples. We denote the misclassified set as $\mathcal {E}=\lbrace \rho \in \mathcal {H}|h(\rho )\ne t(\rho )\rbrace$ and consequently the unitary adversarial set as $\mathcal {E}_{\hat{\epsilon }}=\lbrace \hat{\epsilon }^{-1}(\rho )|\rho \in \mathcal {E}\rbrace$.

**Theorem 2.** For an adversarial perturbation with unitary operator $\hat{\epsilon }$ and *n* samples ρ_1_, …, ρ_*n*_ chosen from $\mathcal {H}$ according to the Haar measure, the performance of the quantum classifier $\mathcal {C}$ with $\hat{\epsilon }(\rho _1),\ldots ,\hat{\epsilon }(\rho _n)$ as input samples is bounded by

(3) \begin{equation*} |R_E-\mu (\mathcal {E})|\le \sqrt{\frac{1}{2n}\ln {\bigg (\frac{2}{\delta }\bigg )}} \end{equation*}

with probability at least 1 − δ (0 < δ < 1). Here *R*_*E*_ is the empirical error rate defined as the ratio of the misclassified samples and $\mu (\mathcal {E})$ is the risk for $\mathcal {C}$. In addition, the expectation of the risk over all ground truth *t* and training set $\mathcal {S}_N$ is bounded below by

(4) \begin{equation*} \mathbb {E}_t[\mathbb {E}_{\mathcal {S}_N}[\mu (\mathcal {E})]]\ge 1-\frac{d^{\prime }}{d(d+1)}(N^2+d+1), \end{equation*}

where $d=\text{dim}(\mathcal {H})$ is the dimension of the input data and *d*^′^ = |*S*| is the number of output labels.

*Proof.* We only sketch the major steps here and leave the details of the full proof to the online supplementary material. Noting that unitary transformations are invertible, the unitary perturbation operator $\hat{\epsilon }$ will transfer samples in $\mathcal {E}_{\hat{\epsilon }}$ into the misclassified set $\mathcal {E}$, and we can therefore deduce that $\mu (\mathcal {E})=\mu (\mathcal {E}_{\hat{\epsilon }})$. Then, from the definition of $\mu (\mathcal {E})$, the inequality in (3) follows straightforwardly by applying Hoeffding’s inequality [48]. The derivation of inequality (4) relies on the recent works about reformulation of the no-free-lunch theorem in the context of quantum machine learning [41,42] (see the online supplementary material for details).

This theorem indicates that, in the limit *d* → ∞, the expectation of the risk for a general quantum classifier goes to unity, independent of its structure and the training algorithm. For a fixed *d*, the lower bound of such an expectation decreases as the number of output labels or the size of the training set increase. Adding an identical adversarial unitary perturbation to all possible data samples will not increase the risk on average. However, it is still possible for such a perturbation to increase the ratio of misclassified samples for a given finite set of *n* original samples. In the following, we carry out numerical simulations and show how to obtain the universal adversarial perturbations in classifying images of handwritten digits and the ground states of the 1D transverse field Ising model. To implement the unitary perturbation $\hat{\epsilon }$, we add an additional variational layer before the original quantum classifiers. After training, we fix the variational parameters of the given classifier $\mathcal {C}$ and optimize the parameters of the perturbation layer through the qBIM algorithm to maximize the loss function for a given set of *n* original samples.

**Figure 3.**
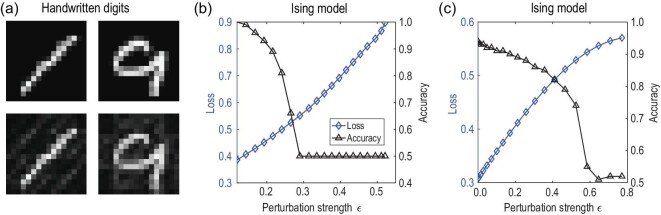
Numerical results on universal adversarial perturbations. Similar to Fig. 2, in this figure the adversarial perturbations are also obtained by the qBIM algorithm with step size α = 0.02. (a) Illustrating samples: the clean (first row) and corresponding adversarial examples (second row) that can fool the second quantum classifier, which is a quantum convolutional neural network. These two adversarial images (second row) are obtained by adding the same perturbation to the original legitimate ones shown in the first row. (b) The loss and accuracy as functions of the perturbation strength ε for the second classifier in classifying the ground states of *H*_Ising_. (c) A similar result for the eighth classifier. Throughout this figure, the white-box attack is considered. For more details, see the online supplementary material.

The major results are shown in Fig. 3. In Fig. 3(a), we display two adversarial examples for digits 1 and 9, which are obtained by adding the same unitary perturbation to the original images and can fool classifier 2 (one of the QCNN classifiers mentioned above). We mention that the fidelity between the original and crafted images is relatively small (about $78\%$) compared with the examples given in Fig. 2(a), but the crafted images remain easily identifiable by human eyes. In Fig. 3(b), we consider adding the same unitary perturbation to all the test samples of the ground states of *H*_Ising_ in a white-box attack setting for classifier 2. From this figure, it is clear that the accuracy drops rapidly at first as we increase the perturbation strength, and then maintains at a fixed finite value (about 0.5). This is consistent with inequality (3) that *R*_*E*_ has an upper bound around $\mu (\mathcal {E})$. We mention that the loss keeps increasing as the perturbation strength increases, even in the region where the accuracy becomes flattened. This counterintuitive behavior is due to the fact that the loss function (cross-entropy) is continuous, whereas the accuracy is defined by the ratio of correctly classified samples whose labels are assigned according to the largest output probability. Fig. 3(c) shows similar results as in Fig. 3(b), but for a different quantum classifier (i.e. classifier 8 mentioned above).

We remark that in our numerical simulations the Hilbert dimension involved is not very large due to limited classical computational resources. Consequently, a larger perturbation is needed to create the adversarial examples. As in Fig. 3(a), the perturbation is perceptible to human eyes. However, this is by no means a pitfall in principle and can be circumvented by simulating larger quantum classifiers. As noisy intermediate-scale quantum devices now become available in laboratories [7], this may also be resolved by running the protocol in real quantum devices. In addition, although we only focus on two-category classifications for simplicity in this paper, the extension to multi-category classifications and other adversarial scenarios is straightforward.

## DISCUSSION AND CONCLUSION

This work only reveals the tip of the iceberg in the fledgling field of quantum adversarial machine learning. Many important questions remain unexplored and demand further investigations. First, this work shows that the existence of universal adversarial examples is a fundamental feature of quantum learning in high-dimensional space in general. However, for a given learning task, the legitimate samples may only occupy a tiny subspace of the whole Hilbert space. This brings about the possibility of defending against adversarial attacks. In practice, how to develop appropriate countermeasures feasible in experiments to strengthen the reliability of quantum classifiers still remains unclear. In addition, unsupervised and reinforcement learning approaches may also suffer from the vulnerability problem [49]. Yet, in practice it is often more challenging to obtain adversarial examples in these scenarios. The study of quantum adversarial learning in the unsupervised or reinforcement setting is still lacking. In particular, how to obtain adversarial examples and perturbations and study their universality properties for quantum unsupervised or reinforcement learning remains entirely unexplored and is well worth future investigations. Finally, it would be interesting and important to carry out an experiment to demonstrate the existence of universal adversarial examples and perturbations. This would be a crucial step toward practical applications of quantum technologies in artificial intelligence in the future, especially for these applications in safety and security-critical environments, such as self-driving cars, malware detection, biometric authentication and medical diagnostics [50].

In summary, we have studied the universality of adversarial examples and perturbations for quantum classifiers. We proved two relevant theorems: one states that an *O*(ln [*k*]/2^*n*^) increase of the perturbation strength is already sufficient to ensure a moderate universal adversarial risk for a set of *k* quantum classifiers, and the other asserts that, for a general quantum classifier, the empirical error rate is bounded from both below and above and approaches unity exponentially fast as the size of the classifier increases. We carried out extensive numerical simulations on concrete examples to demonstrate the existence of universal adversarial examples and perturbations for quantum classifiers in reality. Our results uncover a new aspect of the vulnerability of quantum machine learning systems, which would provide valuable guidance for practical applications of quantum classifiers based on both near-term and future quantum technologies.

## Supplementary Material

nwab130_Supplemental_File
