# Supplementary material for: Universal adversarial examples and perturbations for quantum classifiers
Source: Natl Sci Rev. 2021 Jul 22;9(6):nwab130. doi: 10.1093/nsr/nwab130 (PMC9796671; doi:10.1093/nsr/nwab130)
Supplement: nwab130_Supplemental_File [file nwab130_supplemental_file.pdf]

Supplementary Material for: Universal Adversarial Examples and Perturbations for Quantum Classifiers

Weiyuan Gong[1] and Dong-Ling Deng[1, 2, *]

[1]Center for Quantum Information, IIIS, Tsinghua University, Beijing 100084, People's Republic of China
[2]Shanghai Qi Zhi Institute, 41th Floor, AI Tower, No. 701 Yunjin Road, Xuhui District, Shanghai 200232, China

In this Supplementary Material, we provide more details about the proofs of the two theorems, structures of the quantum classifiers, quantum encoding for classical data, training and attacking processes, and the algorithms for obtaining universal adversarial examples and perturbations.

A. PROOF FOR THEOREM 1

In addition to the ones in the main text, we first give more notations and definitions to formulate the problem.

Definition A1. For $\mathcal{H}' \subseteq \mathcal{H}$, we define the *concentration function* as $\alpha(\epsilon) = 1 - \inf\{\mu(\mathcal{H}'_\epsilon)|\mu(\mathcal{H}) \geq \frac{1}{2}\}$ with distance measure $D(\cdot)$ and probability measure $\mu(\cdot)$ in a d-dimensional vector space. If

$$\alpha(\epsilon) \leq \alpha e^{-\beta\epsilon^2 d}, \tag{S1}$$

then the vector space is said to be in (α, β)-normal Levy group.

We also introduce the following Lemma A1, which has already been obtained in Ref. [1]. Here, we recap the statement and sketch the proof for completeness.

Lemma A1. For a quantum classifier $\mathcal{C}_i$ that takes $\rho \in SU(d)$ according to the Haar measure $\mu(\cdot)$ as input and has a misclassified set $\mathcal{E}_i$. Suppose the adversarial input state ρ' is restricted by $D_{\text{HS}}(\rho, \rho') \leq \epsilon$ to the clean data ρ. Then to guarantee an adversarial risk R_i, ϵ is bounded below by

$$\epsilon^2 \geq \frac{4}{d}\ln[\frac{2}{\mu(\mathcal{E}_i)(1-R_i)}]. \tag{S2}$$

To prove Lemma A1, we further introduce the following two lemmas together with their brief proofs.

Lemma A2. (Theorem 3.7 in [2]) Suppose the input samples are chosen from $\mathcal{H}$ with metric $D(\cdot)$ such that $\mathcal{H}$ is in (α, β)-normal Levy group. For each classifiers $\mathcal{C}_i$ and risk $\mu(\mathcal{E}_i)$, consider additional perturbation $\rho \to \rho'$, $\rho, \rho' \in \mathcal{H}$ and $D(\rho, \rho') \leq \epsilon$. If the adversarial risk $\mu(\mathcal{E}_{i,\epsilon})$ is guaranteed to be at least R_i, then ϵ^2 must also be bounded by

$$\epsilon^2 \geq \frac{1}{\beta d}\ln[\frac{\alpha^2}{\mu(\mathcal{E}_i)(1-R_i)}]. \tag{S3}$$

Proof. We decompose the perturbation $\epsilon = \epsilon_1 + \epsilon_2$. First construct a ϵ_1 such that $\mu(\mathcal{E}_i) > \alpha e^{-\beta\epsilon_1^2 d}$. Consider two cases for whether $\mu(\mathcal{E}_i) \leq \frac{1}{2}$.

(i) If $\mu(\mathcal{E}_i) > \frac{1}{2}$, then we have $\mu(\mathcal{E}_{i,\epsilon_1}) > \mu(\mathcal{E}_i) > \frac{1}{2}$.

(ii) If $\mu(\mathcal{E}_i \leq \frac{1}{2})$, suppose $\mu(\mathcal{E}_{i,\epsilon_1}) \leq \frac{1}{2}$. Then the complement probability $\mu(\mathcal{H}\backslash\mathcal{E}_{i,\epsilon_1}) \geq \frac{1}{2}$. Denote $\mathcal{H}'_i = \mathcal{H}\backslash\mathcal{E}_{i,\epsilon_1}$, then $\mu(\mathcal{H}'_i) \geq \frac{1}{2}$ and $\mathcal{E}_i = \mathcal{H}\backslash\mathcal{H}'_{i,\epsilon_1}$. Hence, we can deduce a contradiction using (S1) as $\alpha(\epsilon_1) \geq 1 - \mu(\mathcal{H}'_{i,\epsilon_1}) = \mu(\mathcal{E}_i) > \alpha(\epsilon_1)$.

Therefore, the perturbation ϵ_1 ensures $\mu(\mathcal{E}_{i,\epsilon_1}) > \frac{1}{2}$. Then we attach ϵ_2 to $\mathcal{E}_{i,\epsilon_1}$, which is $\epsilon = \epsilon_1 + \epsilon_2$ perturbation on $\mathcal{E}_i$. Applying (S1) we can prove the lemma as $R_i = \mu(\mathcal{E}_{i,\epsilon}) = \mu(\mathcal{E}_{i,\epsilon_1+\epsilon_2}) > 1 - \alpha(\epsilon_2)$ and $\epsilon^2 < \epsilon_1^2 + \epsilon_2^2 = \frac{1}{\beta d}\{\ln[\frac{\alpha}{\mu(\mathcal{E}_i)}] + \ln\frac{\alpha}{(1-R_i)}\}$.

Lemma A3. $SU(d)$ group with Haar probability measure and normalized Hilbert-Schmidt metric is in $(\sqrt{2}, \frac{1}{4})$-normal Levy group [3, 4].

Proof. First apply isoperimetric inequality [3, 5], which states that for $\mathcal{H}' \subseteq \mathcal{H}, \dim(\mathcal{H}) = d$ and $\mu(\mathcal{H}') \geq \frac{1}{2}$,

$$\mu(\mathcal{H}'_\epsilon) \geq 1 - \sqrt{2}e^{-\epsilon^2 dR(\mathcal{H})/[2(d-1)]}, \tag{S4}$$

where $R(\mathcal{H}) = \inf_v \text{Ric}(v, v)$ for the Ricci curvature $\text{Ric}(v, v')$ of $\mathcal{H}$ and v goes through all unit tangent vectors in $\mathcal{H}$. Combining (S4) and (S1) we can deduce that

$$\alpha(\epsilon) \leq \sqrt{2}e^{-\epsilon^2 dR(\mathcal{H})/2(d-1)}. \tag{S5}$$

According to [6], for $SU(d)$ equipped with Hilbert-Schmidt metric, $\text{Ric}(v, v) = \frac{d}{2}G(v, v)$. And $G(v, v)$ is the Hilbert-Schmidt metric and v is any unit tangent vector in $SU(d)$. Then from [7] $G(v, v) = 1$. Therefore, $R(\mathcal{H}) = \frac{d}{2}$. This indicates that we can rewrite (S5) as

$$\alpha(\epsilon) \leq \sqrt{2}e^{-\epsilon^2 d^2/4(d-1)} < \sqrt{2}e^{-\epsilon^2 d/4}. \tag{S6}$$

Combining (S3) and (S6), it is shown that for a classifier $\mathcal{C}_i$ with risk $\mathcal{E}_i$ which takes $\rho \in SU(d)$ as input and the Hilbert-Schmidt metric, to bound above adversarial risk with R_i, the adversarial perturbation is bounded below by $\epsilon^2 \geq \frac{4}{d}\ln[\frac{2}{\mu(\mathcal{E}_i)(1-R_i)}]$. Hence, we have completed the proof for Lemma A1.

Now, we continue to prove Theorem 1 in the main text by using the Ineq. (S2). We consider a set of quantum classifiers $\mathcal{C}_i, i = 1, ..., k$ with misclassified set $\mathcal{E}_i, i = 1, ..., k$. Our goal is to calculate $\mu(\mathcal{E}_\epsilon)$ for a given ϵ perturbation. Consider the set $\mathcal{E}_{\text{set}} = \cap_{i=1}^k \mathcal{E}_i$ of original data that is misclassified by all classifiers in the set. If we assume an additional condition $\mathcal{E}_{\text{set}} \neq \emptyset$, then we can construct a quantum classifier $\mathcal{C}^*$ that misclassifies all $\rho \in \mathcal{E}_{\text{set}}$ and correctly classifies other states in $\mathcal{H}$. Then we apply (S2) to this classifier $\mathcal{C}^*$ and can deduce that to guarantee a risk larger than R_0, the perturbation is bounded below by

$$\epsilon^2 \geq \frac{4}{d}\ln[\frac{2}{\mu(\mathcal{E}_{\text{set}})(1-R_0)}]. \tag{S7}$$

If the additional constraint is not satisfied, i.e. $\cap_{i=1}^{k}\mathcal{E}_i = \emptyset$, then we can not directly construct a quantum classifier $\mathcal{C}^*$. In this case, we notice that $\mathcal{E}_\epsilon = \cap_{i=1}^{k}\mathcal{E}_{i,\epsilon} = \mathcal{H} - \cup_{i=1}^{k}(\mathcal{H} - \mathcal{E}_{i,\epsilon})$. Therefore $\mu(\mathcal{E}_\epsilon)$ can be bounded below by:

$$\mu(\mathcal{E}_\epsilon) \geq 1 - \sum_{i=1}^{k} \frac{|\mathcal{H}\backslash\mathcal{E}_{i,\epsilon}|}{|\mathcal{H}|} = \sum_{i=1}^{k}\mu(\mathcal{E}_{i,\epsilon}) - (k-1). \quad \text{(S8)}$$

Hence, if we attach a perturbation that ensures $\mu(\mathcal{E}_{i,\epsilon}) \geq R_{0,i} = \frac{k-1+R}{k}$ for each classifier $\mathcal{C}_i$, then the universal adversarial risk will be bounded below by R. Replacing R and $\mu(\mathcal{E}_i)$ in (S2) with R_0 and $\mu(\mathcal{E})_{\min}$, we finish the proof by arriving at the inequality:

$$\epsilon^2 \geq \frac{4}{d}\ln\left[\frac{2k}{\mu(\mathcal{E})_{\min}(1-R_0)}\right]. \quad \text{(S9)}$$

It is worthwhile to mention that Ineq. (S9) holds regardless of whether the additional assumption $\mathcal{E}_{\text{set}} \neq \emptyset$ is satisfied or not. When $\mathcal{E}_{\text{set}} \neq \emptyset$ is satisfied, the problem reduces to the case for the single classifier $\mathcal{C}^*$. Yet, we cannot tell which inequality, either Ineq. (S7) or (S9), gives a tighter bound as we have no information about the value of $\mu(\mathcal{E}_{\text{set}})$ and $\mu(\mathcal{E}_{\min})$. In our numerical simulations, among the test set containing 100 ground states of the Ising model, we find that there are five samples that can be misclassified by all eight quantum classifiers without adding any perturbation. This indicates that the additional condition might be satisfied in practice.

B. PROOF FOR THEOREM 2

In this section, we provide the details of the proof for Theorem 2 with some further discussions. Following the definitions in the main text, the adversarial operator $\hat{\epsilon}$ is unitary, and hence $\hat{\epsilon}^{-1}$ is also unitary. Then by applying the property of unitary transformation, we have

$$\mu(\mathcal{E}_{\hat{\epsilon}}) = \frac{|\hat{\epsilon}^{-1}(\mathcal{E})|}{|\mathcal{H}|} = \frac{|\mathcal{E}|}{|\mathcal{H}|} = \mu(\mathcal{E}). \quad \text{(S10)}$$

This indicates that the adversarial risk remains the same after we perform the same unitary perturbation operation $\hat{\epsilon}$ on every input quantum state $\rho \in \mathcal{H}$.

We randomly pick $\rho \in \mathcal{H}$ according to the Haar measure. For each selection, the probability of misclassification occurrence is $\mu(\mathcal{E}_{\hat{\epsilon}}) = \mu(\mathcal{E})$. Therefore, we can regard each selection as a random variable, which will be 1 when misclassification occurs and 0 otherwise. Then, we apply Hoeffding's inequality for independent Bernoulli random variables and get with probability at least $1-\delta$ ($\delta > 0$)

$$|R_E - \mu(\mathcal{E})| \leq \sqrt{\frac{1}{2n}\ln\left(\frac{2}{\delta}\right)}. \quad \text{(S11)}$$

This proves the first part of the Theorem 2 in the main text.

To obtain an lower bound for $\mu(\mathcal{E})$, we further resort to the no free lunch theorem [8] and its reformulation in the context of quantum machine learning [9, 10]. Unlike in Ref. [9], where quantum input and output are considered, our discussion is restricted to classification problems in which the output is classical labels. To this end, here we give a loose estimation for the lower bound of $\mu(\mathcal{E})$ with some additional constraints according to our numerical simulations.

In our consideration, the quantum classifier takes two steps to classify input samples. In the first step, the classifier takes a quantum state $\rho \in \mathcal{H}$ as input and undergoes a variational circuit to arrive at the output state ρ_{out} belonging to a d'-dimensional Hilbert space. In the second step, the classifier outputs a label $s \in \{0, 1, ..., d'-1\}$ according to the largest probability among $\langle 0|\rho_{\text{out}}|0\rangle, \langle 1|\rho_{\text{out}}|1\rangle, ..., \langle d'-1|\rho_{\text{out}}|d'-1\rangle$. Based on this, our analysis of $\mu(\mathcal{E})$ will lead to an average performance bound for the classifier [9].

In the first step from ρ to ρ_{out}, the quantum ground truth is defined as a unitary process t. Without loss of generality, we may restrict our discussion to the case of quantum pure states. The training set is rewritten as $\mathcal{S}_N = \{(|\psi_1\rangle, |\phi_1\rangle), ..., (|\psi_N\rangle, |\phi_N\rangle)\}$ and the classifier learns a hypothesis operator V, which is a unitary process such that $t|\psi_i\rangle = V|\psi_i\rangle = |\phi_i\rangle$ for the training set. The quantum risk function is defined as [11].

$$R_t(V) \equiv \int d|\psi\rangle ||t|\psi\rangle\langle\psi|t^\dagger - V|\psi\rangle\langle\psi|V^\dagger||_1^2, \quad \text{(S12)}$$

where $||A||_1$ is the trace norm for matrices [12]. Now the quantum no free lunch theorem is described as below.

*Lemma B*1.*(Quantum No Free Lunch)* The quantum risk function in a classification task averaged over selection of quantum ground truth t and training set S_N with respect to the Haar measure can be bounded below by

$$\mathbb{E}_t[\mathbb{E}_{\mathcal{S}_N}[R_t(V)]] \geq 1 - \frac{1}{d(d+1)}(N^2 + d + 1). \quad \text{(S13)}$$

The proof of this lemma and more discussions about its implications are provided in Refs. [9, 10]. Here, we use this lemma to obtain Ineq. (4) in the main text. Noting that $||A||_1 \leq 1$, hence for all the $\rho = |\psi\rangle\langle\psi| \in \mathcal{E}$, $D(t|\psi\rangle, V|\psi\rangle) = ||t|\psi\rangle\langle\psi|t^\dagger - V|\psi\rangle\langle\psi|V^\dagger||_1 \leq 1$. This means that $R_t(V) \leq 1$, regardless of whether the quantum data is correctly classified or not.

Then we come to the case when a quantum input is classified correctly. Without loss of generality, we can assume that the ground truth gives true label and output state $t|\psi\rangle = |i\rangle$, then since the quantum data is correctly predicted, $\langle i|t|\psi\rangle\langle\psi|t^\dagger|i\rangle \geq \frac{1}{d'}$. From this inequality, we obtain that the fidelity $F(V|\psi\rangle, t|\psi\rangle = |i\rangle) \geq \sqrt{\frac{1}{d'}}$. We can utilize the relation between fidelity and the trace norm

$$D(\rho, \sigma)^2 \leq 1 - F(\rho, \sigma)^2, \quad \text{(S14)}$$

where ρ, σ denote arbitrary quantum states.

Hence, for correctly classified quantum data we have $R_t(V) = D(t|\psi\rangle, V|\psi\rangle)^2 = ||t|\psi\rangle\langle\psi|t^\dagger - V|\psi\rangle\langle\psi|V^\dagger||_1^2 \leq$

$1 - F(t|\psi\rangle, V|\psi\rangle)^2 \leq 1 - \frac{1}{d'}$. As a result, the integral in Eq. (S12) is bounded by

$$R_t(V) \leq \mu(\mathcal{E}) + \frac{d'-1}{d'}(1-\mu(\mathcal{E})) = \frac{1}{d'}(d'-1+\mu(\mathcal{E})). \tag{S15}$$

Combining (S13) and (S15), we obtain a lower bound of $\mu(\mathcal{E})$ averaged over ground truth t and training set $\mathcal{S}_N$

$$\mathbb{E}_t[\mathbb{E}_{\mathcal{S}_N}[\mu(\mathcal{E})]] \geq 1 - \frac{d'}{d(d+1)}(N^2+d+1). \tag{S16}$$

This completes the proof of Theorem 2.

C. THE STRUCTURES OF QUANTUM CLASSIFIERS AND ENCODING METHODS

I. The structures of quantum classifiers

In recent years, a number of different quantum classifiers have been proposed [13–28]. Here, we choose some of these classifiers to form the classifier set considered in this paper. As mentioned in the main text, our classifier set contains two QCNNs [21] and six general multi-layer variational classifiers [13, 14, 16, 17]. The sketch of a quantum variational circuit is shown in Fig. S1(a).

In such a variational circuit model, we first prepare the $(m+n)$-qubit input state to be $|\psi_{\text{in}}\rangle \otimes |1\rangle^{\otimes m}$, where $|\psi\rangle_{\text{in}}$ is an n-qubit state that encodes the complete information of input sample to be classified. Then we apply a unitary transformation, which is composed of p layers of interleaved operations, on the state. In each of the p layers, there are two rotation units each performs arbitrary Euler rotations in Bloch sphere and an entangler unit consisting of CNOT gates between each pair of neighboring qubits. The adjustable parameters are the rotation angles and are collectively denoted as Θ. This generates a variational state:

$$|\Phi(\Theta)\rangle = \prod_{i=1}^{p} U_i(|\psi_{\text{in}}\rangle \otimes |1\rangle^{\otimes m}), \tag{S17}$$

where $U_i = \prod_k Z(\theta^k_{i,d})X(\theta^k_{i,c})U_{\text{ent}}Z(\theta^k_{i,b})X(\theta^k_{i,a})$ denotes the unitary operation for the i-th layer, with U_{ent} representing the unitary operation generated by the entangler unit.

The brief structure of the QCNN and its hyperparameters utilized in this paper is shown in Fig. S1(b). The structure of the QCNN is the same as in Ref. [21].

In our numerical simulations, we only focus on two-category classification problems. Thus, we only need one qubit to encode the labels $y = 0, 1$. After the variational circuits, the state of the output qubits becomes ρ_{out}. We compute $\mathbb{P}(y = m) = \text{Tr}(\rho_{\text{out}}|m\rangle\langle m|)$ and then assign $y = 1$ if $P(y = 1) \geq P(y = 0)$ and $y = 0$ for other cases.

II. Quantum encoding for classical data

In the main text, one of the numerical simulations we did is based on the images of handwritten digits. In this dataset, the images are encoded classically, i.e. the data is encoded into a m-dimensional vector $\mathbf{v}$ in $\mathbb{R}^m$. To make such classical data processable to quantum classifiers, we need to convert the classic vector into a n-qubit quantum (pure) state in a $d = 2^n$ dimensional Hilbert space. This converting process is called a quantum encoder. In this paper, we use the amplitude encoder to transfer classical data into quantum states [13, 15, 26, 29–36].

For an amplitude encoder, each component of $\mathbf{v}$ is then represented by the amplitude of the n-qubit ket vector $|\psi_{\text{in}}\rangle$ represented in computational basis. Without loss of generality, we assume that $m = 2^n$ is a power of 2, otherwise we can attach $2^n - m$ zeros to the end of the vector $\mathbf{v}$ so that it can be transformed into a n-qubit pure state. The encoder can be realized by a circuit and the depth of the circuit is linear with the number of features [37–39]. Under certain conditions, a polynomial size of gate complexity over m might be needed [40, 41]. Such encoding procedure can be improved using a more complex approach like tensorial feature maps [13].

III. The training process of quantum classifiers

In classical machine learning, different loss functions are introduced when training the networks and estimating the performance. In numerical simulations, we employ a quantum version of cross-entropy as

$$\mathcal{L}(h(|\psi\rangle;\Theta), \mathbf{p}) = -\sum_{i=1}^{2} p_k \log q_k, \tag{S18}$$

where $\mathbf{q} = (q_1, q_2)$ is the diagonalized expression of output state diag(ρ_{out}) and $\mathbf{p} = (1, 0)$ for $y = 0$ and $\mathbf{p} = (0, 1)$ for $y = 1$. In the training procedure of a quantum classifier, an optimizer is used to adjust the parameter Θ to minimize the empirical loss function $\mathcal{L}_N(\theta) = \frac{1}{N}\sum_{i=1}^{N}\mathcal{L}(h(|\psi_i\rangle;\Theta), \mathbf{p}_i)$. In recent years, a large family of gradient-based algorithms have been broadly used in training classical and quantum neural networks [42–46]. In the numerical simulations in this research, we use Adam optimization algorithm [45, 46], which is a gradient-based learning algorithm with adaptive learning rate.

To find the minimization of the loss function using multiple-step gradient-based methods, we need to calculate the gradient of $\mathcal{L}_N(\Theta)$ over parameter Θ. Each component of the gradient is represented as $\frac{\partial \mathcal{L}_N(\Theta)_\theta}{\partial \theta} = \lim_{\epsilon\to 0}\frac{1}{2\epsilon}[\mathcal{L}_N(\Theta)_{\theta+\epsilon} - \mathcal{L}_N(\Theta)_{\theta-\epsilon}]$ where θ is one of the parameters of Θ. Owing to the special structures of the quantum classifiers, we use the ”parameter shift rule” [47–49] in our numerical simulations to obtain the gradients required.

In Fig. S2, we plot the average loss and accuracy of some of quantum classifiers in our classifier set during the training pro-

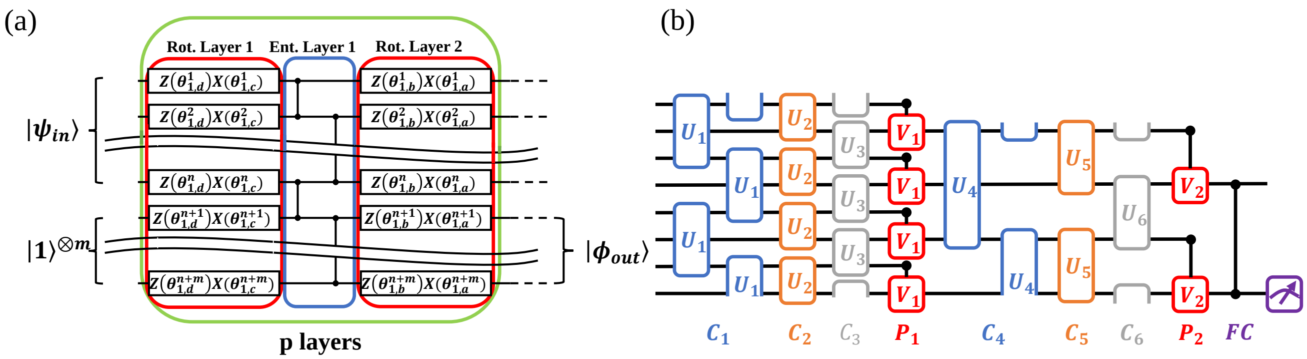

FIG. S1. The structure of quantum classifiers used in the numerical simulations. (a)The illustrative structure of a general multi-layer quantum variational classifier that takes n-qubit state $|\psi_{\text{in}}\rangle$ as input and outputs a m-qubit state $|\phi_{\text{out}}\rangle$. The classifier consists of p layers and each layer consists two rotation units and an entangler unit. Each rotation unit contains a Euler rotation $Z(\theta^k_{i,u})X(\theta^k_{i,v})$ $[(u,v)=(d,c)$ or $(b,a)]$, where $i=1,...,p$ refer to the number of layers, $k=1,...,m+n$ denotes the number of qubit. After obtaining the output state $|\phi_{\text{out}}\rangle$, we compute the probabilities of projection measurements to predict and assign a label that corresponds to the largest probability. (b) The illustrative structure of the QCNN classifier. This circuit contains six convolutional layers labeled by C_1 to C_6, two polling layers labeled by P_1 and P_2 respectively, and a fully connected layer labeled by FC. The initial parameters are set to random values at the beginning of the training process.

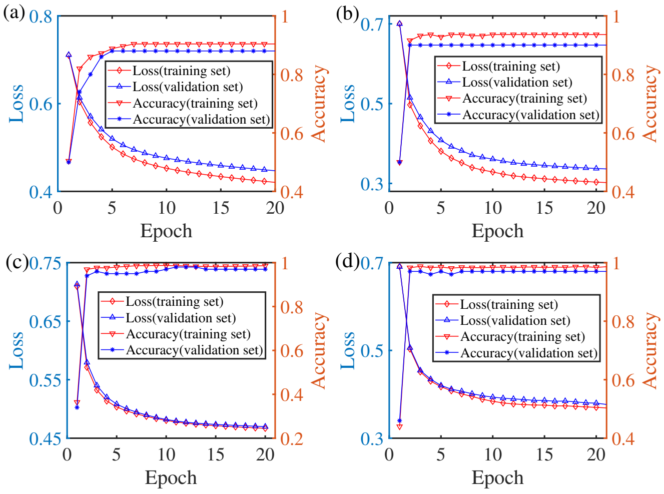

FIG. S2. The average loss and accuracy for quantum classifiers 2 and 8 during the training process, in classifying handwritten digit images and the ground states of the 1D transverse field Ising model. (a) The training procedure of the classifier 2 (a QCNN classifier) for classifying the ground states. Each epoch contains 30 iterations. (b) The training procedure of the classifier 8 with depth $p=10$ for classifying the ground states. Each epoch contains 5 iterations. (c) The training procedure of the classifier 2 for classifying handwritten digit images. Each epoch contains 50 iterations. (d)The training procedure of classifier 8 for classifying handwritten digit images. Each epoch represents 10 iterations.

cedure. The numerical simulations including the training procedure and adversarial attack were done on a classical cluster using Yao.jl [50] and its extension packages in Julia language [51]. To run the simulation on GPU, i.e. to perfectly fit the mini-batch gradient descent algorithm, we use CuYao.jl [52]. This package is an efficient extension of Yao.jl on GPU calculation that can obtain a speedup. Flux.jl [53] and Zygote.jl [54] packages are used to calculate the differentiation of the function. We note that the overfitting risk is low as the loss of the training data and validation data is close [55].

In Table S1, we list the number of parameters for each quantum classifier used in this paper, and their final accuracy in classifying the ground states of the 1D transverse field Ising model.

Classifier	Structure	Number of parameters	Accuracy
1	QCNN	44	0.917
2	QCNN	92	0.950
3	Variational Circuit	144	0.923
4	Variational Circuit	171	0.930
5	Variational Circuit	198	0.940
6	Variational Circuit	225	0.930
7	Variational Circuit	252	0.947
8	Variational Circuit	279	0.955

TABLE S1. The number of parameters and the final accuracy after the training process for each quantum classifier in classifying the ground states of the 1D Ising model. The accuracy is calculated over a training set that contains 300 samples.

D. ADVERSARIAL ALGORITHMS

In this section, we provide more details on the algorithms for obtaining adversarial examples and perturbations.

When proposing an adversarial attack on a quantum classifier that takes quantum data as input, we maximize the adversarial risk $\mu(\mathcal{E})$ mentioned in the main text. However, in practice $\mu(\mathcal{E})$ is typically inaccessible. Hence, we consider maximizing the loss function instead. It is worthwhile to mention that a maximal loss function value does not always indicates a maximal risk. In the quantum scenario, we denote the adversarial perturbation attached to the quantum sample as an

operator U_δ that acts on the given input state. The maximization problem of adding perturbation can be described as:

$$U_\delta \equiv \underset{U_\delta \in \Delta}{\arg\max}\, \mathcal{L}(h(U_\delta|\psi\rangle;\Theta^*),\mathbf{p}), \tag{S19}$$

where Θ^* denotes the optimized parameters after the training process, Δ are the possible perturbations that can be added, $|\psi\rangle$ is the original input state and $\mathbf{p}$ is the correct label. In the case of studying universal adversarial examples, we have a test set $\mathcal{T}_M = \{(|\psi_0\rangle, y_0), ..., (|\psi_M\rangle, y_M)\}$ and a set of quantum classifiers which learn hypothesis functions $h_1, h_2, ..., h_k$. In order to obtain universal adversarial examples that can deceive all the quantum classifiers in the set, we solve the following optimization problem:

$$U_\delta^j \equiv \underset{U_\delta^j \in \Delta}{\arg\max} \sum_{i=1}^{k} \mathcal{L}(h_i(U_\delta^j|\psi_j\rangle;\Theta^*),\mathbf{p}_j), \tag{S20}$$

where U_δ^j is the perturbation for the j-th sample. For the case of obtaining universal adversarial perturbations, we use an identical perturbation to implement the adversarial attack to all samples in the test set $\mathcal{T}_M$. In this case, we have one quantum classifier and its hypothesis function h. The maximization problem can be expressed in the similar form

$$U_\delta \equiv \underset{U_\delta \in \Delta}{\arg\max} \frac{1}{M}\sum_{i=1}^{M} \mathcal{L}(h(U_\delta|\psi_i\rangle;\Theta^*),\mathbf{p}_i). \tag{S21}$$

In general, the set Δ can be the set of unitary operators that are close to the identity matrix. We use automatic differentiation [56] to improve precision when applying the perturbation. In practice, we restrict the set Δ to be the product of local unitary operators near the identity matrix.

In the white-box attack scenario, the attacker has the full information of the classifiers, including their inner structures and loss functions. The attacker can then calculate the gradient of loss functions $\nabla\mathcal{L}(h(|\psi\rangle;\Theta^*),\mathbf{p})$. In this scenario, we use the quantum-adapted Basic Iterative Method (qBIM) introduced in Ref. [49] to solve the above optimization problems in Eq. (S20) and (S21).

Compared with a white-box scenario, the adversary in a black-box setting does not have complete information of the quantum classifier. In classical adversarial learning, the development of black-box assumption has been divided into several categories. In non-adaptive black-box attack [57–59], the adversary knows nothing about the classifier's inner structure but can get access to the training data and analyze its distribution. In adaptive black-box scenario [58, 60, 61], the attacker can use the classifier as an oracle without extra information provided. Another category is the strict black-box scenario [62], where the data distribution is unknown but the adversary can collect the input-output pairs from the target classifier. In our simulations, we implement the non-adaptive black-box adversarial attack in which we try to use the knowledge of one quantum classifier to attack all quantum classifiers in the set that share the same training set and test set. The result shown in Fig. 2(c) in the main text indicates the effectiveness of such a black-box attack.

* dldeng@tsinghua.edu.cn

[1] N. Liu and P. Wittek, "Vulnerability of quantum classification to adversarial perturbations," Phys. Rev. A **101**, 062331 (2019).

[2] S. Mahloujifar, D. I. Diochnos, and M. Mahmoody, "The curse of concentration in robust learning: Evasion and poisoning attacks from concentration of measure," in *Proceedings of the AAAI Conference on Artificial Intelligence*, Vol. 33 (2019) pp. 4536–4543.

[3] M. Gromov and V. D. Milman, "A topological application of the isoperimetric inequality," Am. J. Math. **105**, 843 (1983).

[4] T. Giordano and V. Pestov, "Some extremely amenable groups related to operator algebras and ergodic theory," J. Inst. Math. Jussieu **6**, 279 (2007).

[5] V. D. Milman and G. Schechtman, *Asymptotic theory of finite dimensional normed spaces: Isoperimetric inequalities in riemannian manifolds*, Vol. 1200 (Springer, 2009).

[6] E. Meckes, *Concentration of measure and the compact classical matrix groups*, edited by (unpublished) (Citeseer, 2014).

[7] M. Oszmaniec, R. Augusiak, C. Gogolin, J. Kołodyński, A. Acin, and M. Lewenstein, "Random bosonic states for robust quantum metrology," Phys. Rev. X **6**, 041044 (2016).

[8] S. Shalev-Shwartz and S. Ben-David, *Understanding machine learning: From theory to algorithms* (Cambridge university press, 2014).

[9] K. Poland, K. Beer, and T. J. Osborne, "No free lunch for quantum machine learning," arXiv:2003.14103 (2020).

[10] K. Sharma, M. Cerezo, Z. Holmes, L. Cincio, A. Sornborger, and P. J. Coles, "Reformulation of the no-free-lunch theorem for entangled data sets," arXiv:2007.04900 (2020).

[11] A. Monras, G. Sentís, and P. Wittek, "Inductive supervised quantum learning," Phys. Rev. Lett. **118**, 190503 (2017).

[12] M. A. Nielsen and I. L. Chuang, *Quantum Computation and Quantum Information* (Cambridge University Press, Cambridge, 2010).

[13] M. Schuld, A. Bocharov, K. M. Svore, and N. Wiebe, "Circuit-centric quantum classifiers," Phys. Rev. A **101**, 032308 (2020).

[14] E. Farhi and H. Neven, "Classification with quantum neural networks on near term processors," arXiv:1802.06002 (2018).

[15] M. Schuld, M. Fingerhuth, and F. Petruccione, "Implementing a distance-based classifier with a quantum interference circuit," EPL (Europhysics Letters) **119**, 60002 (2017).

[16] K. Mitarai, M. Negoro, M. Kitagawa, and K. Fujii, "Quantum circuit learning," Phys. Rev. A **98**, 032309 (2018).

[17] J. Li, X. Yang, X. Peng, and C.-P. Sun, "Hybrid quantum-classical approach to quantum optimal control," Phys. Rev. Lett. **118**, 150503 (2017).

[18] M. Schuld and N. Killoran, "Quantum machine learning in feature hilbert spaces," Phy. Rev. Lett. **122**, 040504 (2019).

[19] V. Havlíček, A. D. Córcoles, K. Temme, A. W. Harrow, A. Kandala, J. M. Chow, and J. M. Gambetta, "Supervised learning with quantum-enhanced feature spaces," Nature **567**, 209 (2019).

[20] D. Zhu, N. M. Linke, M. Benedetti, K. A. Landsman, N. H. Nguyen, C. H. Alderete, A. Perdomo-Ortiz, N. Korda, A. Garfoot, C. Brecque, *et al.*, "Training of quantum circuits on a hybrid quantum computer," Sci. Adv. **5**, eaaw9918 (2019).

[21] I. Cong, S. Choi, and M. D. Lukin, "Quantum convolutional

neural networks," Nat. Phys. **15**, 1273 (2019).
[22] K. H. Wan, O. Dahlsten, H. Kristjánsson, R. Gardner, and M. Kim, "Quantum generalisation of feedforward neural networks," npj Quantum Inf. **3**, 1 (2017).
[23] E. Grant, M. Benedetti, S. Cao, A. Hallam, J. Lockhart, V. Stojevic, A. G. Green, and S. Severini, "Hierarchical quantum classifiers," npj Quantum Inf. **4**, 1 (2018).
[24] Y. Du, M.-H. Hsieh, T. Liu, and D. Tao, "Implementable quantum classifier for nonlinear data," arXiv:1809.06056 (2018).
[25] A. Uvarov, A. Kardashin, and J. D. Biamonte, "Machine learning phase transitions with a quantum processor," Phys. Rev. A **102**, 012415 (2020).
[26] P. Rebentrost, M. Mohseni, and S. Lloyd, "Quantum support vector machine for big data classification," Phys. Rev. Lett. **113**, 130503 (2014).
[27] C. Blank, D. K. Park, J.-K. K. Rhee, and F. Petruccione, "Quantum classifier with tailored quantum kernel," npj Quantum Inf. **6**, 1 (2020).
[28] F. Tacchino, C. Macchiavello, D. Gerace, and D. Bajoni, "An artificial neuron implemented on an actual quantum processor," npj Quantum Inf. **5**, 1 (2019).
[29] A. W. Harrow, A. Hassidim, and S. Lloyd, "Quantum algorithm for linear systems of equations," Phys. Rev. Lett. **103**, 150502 (2009).
[30] I. Cong and L. Duan, "Quantum discriminant analysis for dimensionality reduction and classification," New J. Phys. **18**, 073011 (2016).
[31] I. Kerenidis and A. Prakash, "Quantum recommendation systems," in *8th Innovations in Theoretical Computer Science Conference (ITCS 2017)* (Schloss Dagstuhl-Leibniz-Zentrum fuer Informatik, 2017).
[32] V. Giovannetti, S. Lloyd, and L. Maccone, "Architectures for a quantum random access memory," Phys. Rev. A **78**, 052310 (2008).
[33] S. Lloyd, M. Mohseni, and P. Rebentrost, "Quantum algorithms for supervised and unsupervised machine learning," arXiv:1307.0411 (2013).
[34] N. Wiebe, A. Kapoor, and K. M. Svore, "Quantum deep learning," arXiv:1412.3489 (2014).
[35] V. Giovannetti, S. Lloyd, and L. Maccone, "Quantum random access memory," Phys. Rev. Lett. **100**, 160501 (2008).
[36] A. Scott, "Read the fine print," Nat. Phys. **11**, 291 (2015).
[37] M. Möttönen, J. J. Vartiainen, V. Bergholm, and M. M. Salomaa, "Quantum circuits for general multiqubit gates," Phys. Rev. Lett. **93**, 130502 (2004).
[38] E. Knill, "Approximation by quantum circuits," arXiv:quant-ph/9508006 (1995).
[39] M. Plesch and Č. Brukner, "Quantum state preparation with universal gate decompositions," Phys. Rev. A **83**, 032302 (2011).
[40] L. Grover and T. Rudolph, "Creating superpositions that correspond to efficiently integrable probability distributions," arXiv: quant-ph/0208112 (2002).
[41] A. N. Soklakov and R. Schack, "Efficient state preparation for a register of quantum bits," Phys. Rev. A **73**, 012307 (2006).
[42] F. Wilde, R. Sweke, J. Meyer, M. Schuld, P. Fährmann, B. Meynard-Piganeau, and J. Eisert, "Stochastic gradient descent for hybrid quantum-classical optimization," Bull. Am. Phys. Soc. **65** (2020).
[43] N. Yamamoto, "On the natural gradient for variational quantum eigensolver," arXiv:1909.05074 (2019).
[44] J. Stokes, J. Izaac, N. Killoran, and G. Carleo, "Quantum natural gradient," arXiv:1909.02108 (2019).
[45] D. P. Kingma and J. Ba, "Adam: A method for stochastic optimization," arXiv:1412.6980 (2014).
[46] J. R. Sashank, K. Satyen, and K. Sanjiv, "On the convergence of adam and beyond," in *6th International Conference on Learning Representations, ICLR 2018, Vancouver, BC, Canada, April 30 - May 3, 2018, Conference Track Proceedings* (2018).
[47] J.-G. Liu and L. Wang, "Differentiable learning of quantum circuit born machines," Phys. Rev. A **98**, 062324 (2018).
[48] A. Harrow and J. Napp, "Low-depth gradient measurements can improve convergence in variational hybrid quantum-classical algorithms," arXiv:1901.05374 (2019).
[49] S. Lu, L.-M. Duan, and D.-L. Deng, "Quantum adversarial machine learning," Phys. Rev. Res. **2**, 033212 (2020).
[50] "https://github.com/quantumbfs/ yao.jl," .
[51] J. Bezanson, A. Edelman, S. Karpinski, and V. B. Shah, "Julia: A fresh approach to numerical computing," SIAM review **59**, 65 (2017).
[52] "https://github.com/quantumbfs/cuyao.jl," .
[53] M. Innes, "Flux: Elegant machine learning with julia," J. Open Source Softw. **3**, 602 (2018).
[54] "https://github.com/fluxml/zygote.jl," .
[55] N. Srivastava, G. Hinton, A. Krizhevsky, I. Sutskever, and R. Salakhutdinov, "Dropout: a simple way to prevent neural networks from overfitting," J. Mach. Learn. Res. **15**, 1929 (2014).
[56] L. B. Rall and G. F. Corliss, "An introduction to automatic differentiation," Computational Differentiation: Techniques, Applications, and Tools **89** (1996).
[57] N. Papernot, P. McDaniel, and I. Goodfellow, "Transferability in machine learning: from phenomena to black-box attacks using adversarial samples," arXiv:1605.07277 (2016).
[58] N. Papernot, P. McDaniel, I. Goodfellow, S. Jha, Z. B. Celik, and A. Swami, "Practical black-box attacks against machine learning," in *Proceedings of the 2017 ACM on Asia conference on computer and communications security* (2017) pp. 506–519.
[59] F. Tramèr, F. Zhang, A. Juels, M. K. Reiter, and T. Ristenpart, "Stealing machine learning models via prediction apis," in *25th {USENIX} Security Symposium ({USENIX} Security 16)* (2016) pp. 601–618.
[60] M. Fredrikson, S. Jha, and T. Ristenpart, "Model inversion attacks that exploit confidence information and basic countermeasures," in *Proceedings of the 22nd ACM SIGSAC Conference on Computer and Communications Security* (2015) pp. 1322–1333.
[61] I. Rosenberg, A. Shabtai, L. Rokach, and Y. Elovici, "Generic black-box end-to-end attack against rnns and other api calls based malware classifiers," arXiv:1707.05970 (2017).
[62] B. Hitaj, G. Ateniese, and F. Perez-Cruz, "Deep models under the gan: information leakage from collaborative deep learning," in *Proceedings of the 2017 ACM SIGSAC Conference on Computer and Communications Security* (2017) pp. 603–618.
